# Recombinant Adeno-Vaccine Expressing Enterovirus 71-Like Particles against Hand, Foot, and Mouth Disease

**DOI:** 10.1371/journal.pntd.0003692

**Published:** 2015-04-09

**Authors:** Yueh-Liang Tsou, Yi-Wen Lin, Hsiao-Yun Shao, Shu-Ling Yu, Shang-Rung Wu, Hsiao-Yu Lin, Chia-Chyi Liu, Chieh Huang, Pele Chong, Yen-Hung Chow

**Affiliations:** 1 Institute of Infectious Disease and Vaccinology, National Health Research Institutes, Zhunan, Taiwan; 2 Graduate Program of Biotechnology in Medicine, Institute of Molecular and Cellular Biology, National Tsing Hua University, Hsinchu, Taiwan; 3 Institute of Oral Medicine, National Cheng Kung University College of Medicine and Hospital, Tainan, Taiwan; 4 Graduate Institute of Immunology, China Medical University, Taichung, Taiwan; Florida Gulf Coast University, UNITED STATES

## Abstract

Enterovirus 71 (EV71) and coxsackieviruses (CV) are the major causative agents of hand, foot and mouth disease (HFMD). There is not currently a vaccine available against HFMD, even though a newly developed formalin-inactivated EV71 (FI-EV71) vaccine has been tested in clinical trial and has shown efficacy against EV71. We have designed and genetically engineered a recombinant adenovirus Ad-EVVLP with the EV71 *P1* and *3CD* genes inserted into the E1/E3-deleted adenoviral genome. Ad-EVVLP were produced in HEK-293A cells. In addition to Ad-EVVLP particles, virus-like particles (VLPs) formed from the physical association of EV71 capsid proteins, VP0, VP1, and VP3 expressed from *P1* gene products. They were digested by 3CD protease and confirmed to be produced by Ad-EVVLP-producing cells, as determined using transmission electron microscopy and western blotting. Mouse immunogenicity studies showed that Ad-EVVLP-immunized antisera neutralized the EV71 B4 and C2 genotypes. Activation of VLP-specific CD4^+^ and CD8^+^/IFN-γ T cells associated with Th1/Th2-balanced IFN-ɣ, IL-17, IL-4, and IL-13 was induced; in contrast, FI-EV71 induced only Th2-mediated neutralizing antibody against EV71 and low VLP-specific CD4^+^ and CD8^+^ T cell responses. The antiviral immunity against EV71 was clearly demonstrated in mice vaccinated with Ad-EVVLP in a hSCARB2 transgenic (hSCARB2-Tg) mouse challenge model. Ad-EVVLP-vaccinated mice were 100% protected and demonstrated reduced viral load in both the CNS and muscle tissues. Ad-EVVLP successfully induced anti-CVA16 immunities. Although antisera had no neutralizing activity against CVA16, the 3C-specific CD4^+^ and CD8^+^/IFN-γ T cells were identified, which could mediate protection against CVA16 challenge. FI-EV71 did not induce 3C-mediated immunity and had no efficacy against the CVA16 challenge. These results suggest that Ad-EVVLP can enhance neutralizing antibody and protective cellular immune responses to prevent EV71 infection and cellular immune responses against CV infection.

## Author Summary

The spread of enterovirus-induced HFMD could be controlled through a robust vaccination program. Formalin-inactivated EV71 (FI-EV71) vaccines have been evaluated in human clinical trials in China, Taiwan and Singapore and were found to be safe and to elicit strong neutralizing antibody responses against EV71, which is currently circulating in Asia. However, the results from recent three phase III clinical trials performed in young children indicate that the lack of efficacy against CVA16 infections is a major challenge to the current EV71 vaccine and future HFMD vaccine development. In this study, we developed an adenovirus-based vaccine, Ad-EVVLP with the EV71 *P1* and *3CD* genes to express VLPs. Ad-EVVLP immunization induced EV71-specific neutralizing antibodies and Th1/Th2-balanced cellular responses in mice. Ad-EVVLP provided protection against both EV71 and CVA16 challenges in the hSCARB2-Tg mice model, whereas FI-EV71 vaccine activated only Th2-mediated EV71 neutralizing antibody responses to protect against EV71 challenge. Because Ad-EVVLP vaccination-induced antibodies had no virus neutralizing activities against CVA16, the cross-protective immunity against CVA16 was mediated by conserved 3CD-specific cellular immunity activation. These results indicate that Ad-EVVLP meets a medical need as a universal HFMD vaccine against both EV71 and CV infections.

## Introduction

Enterovirus 71 (EV71) and coxsackievirus (CVA) infections are the most common causative factors of hand, foot, and mouth disease (HFMD) and other neurological disorders. Severe neurological disorders, including encephalitis, acute flaccid paralysis, pulmonary edema (PE), and hemorrhaging, culminating in fatality, particularly in EV71-infected children under 5 years old, have been reported [1–5]. Because HFMD and EV71 infections can potentially become a new threat to global public health [1,6–11], effective antiviral drugs and prophylactic vaccines are urgently needed.

Enterovirus is a nonenveloped, single-stranded RNA virus of the *Picornaviridae* family. Its genome consists of a single open reading frame that encodes the P1, P2, and P3 poly-proteins. The P2 and P3 regions encode nonstructural proteins (e.g., 3CD) responsible for virus replication and virulence. During viral RNA translation, the 2A protein catalyzes its N-terminal cleavage in cis, thereby releasing the capsid proteins in the P1 region from the nascent nonstructural proteins in the P2 and P3 regions. 3CD is released from the P3 precursor by autocatalytic cleavage. A 3C’ cleavage site in the polyprotein resides between the 3C and 3D portion of 3CD to generate 2 products, 3C’ and 3D’. When the P1 precursor is encoded by the P1 region, it can be cleaved by the 3C’ protease into VP0, VP1, and VP3. These 3 proteins spontaneously assemble into an icosahedral procapsid and pack the RNA genome into the provirion that could be a non-infectious empty (E)-particle or infectious full (F)-particle [12,13].

Human scavenger receptor class B, member 2 (hSCARB2) and human P-selectin glycoprotein ligand 1 (PSGL-1) have been identified as the important cell receptors for EV71 infection [14,15]. Our group [16] and Fujii *et al*. [17] have successfully developed transgenic mice expressing the human hSCARB2 receptor. In this promising model, transgenic animals infected with clinical EV71 isolates of the B4 and B5 subgenotypes developed HFMD-like skin rashes, whereas those inoculated with EV71 C2 and C4 subgenotypes or CVA16 suffered severe limb paralysis and death. In addition, passive administration of the monoclonal anti-EV71 VP1 neutralizing antibody N3 [18] reduced EV71 B5 infection-induced symptoms and protected the transgenic mice against EV71 C2-induced severe limb paralysis and death[19].

In a previous study[20], we produced a formalin-inactivated EV71 strain E59 (FI-EV71) vaccine candidate formulated with alum adjuvant, and found that FI-EV71 displayed high efficacy in the hSCARB2-Tg mouse challenge model [16]. In a human phase I clinical trial[21], FI-EV71 was safe and could elicit strong neutralizing antibody responses against current circulating EV71 isolates, but failed to protect against CVA16 infections. A replication-incompetent adenovirus (Ad) containing a fusion protein (F) gene of the respiratory syncytia virus (RSV) could induce viral antigen specific neutralizing antibody and cellular immunity against RSV infection in a mouse model [22]. In addition, Ad-expressing human immunodeficiency virus gp120 was tested as a vaccine in human trials and was confirmed to be safe [23]. However, some reports have argued that preexisting anti-adenovirus antibodies can influence the efficacy of the intramuscular injection of Ad vaccine intramuscular in clinical use [23]. Previous studies have shown that preexisting antibodies do not affect the subsequent generation of humoral responses to the antigen expressed by a recombinant Ad when administered mucosally [24,25].

In this study, we designed a recombinant Ad-5 carrying both *P1* and *3CD* genes of EV71 (Ad-EVVLP). Upon Ad-EVVLP replication in HEK-293A cells, EV71 VLPs could self-assemble through the cleavage of P1 polyprotein into individual proteins (VP1, VP3, and VP0) by the 3C’ protease. We investigated the immunogenicity of Ad-EVVLP in mice to determine whether neutralizing antibodies and Th1/Th2 cellular responses were induced to cross-protect against EV71 and CVA16 in animal models.

## Materials and Methods

### Ethics statement

All animal experiments were conducted in accordance with the guidelines of the Laboratory Animal Center of the National Health Research Institutes (NHRI), Taiwan. Animal use protocols were reviewed and approved by the NHRI Institutional Animal Care and Use Committee (Approval Protocol No. NHRI-IACUC-100125-A). In EV71 challenge experiments, survival rate was used as an endpoint to assess the protective efficacy of the anti-EV71 treatment. Survival rate used as an index of pathogenesis of EV71 infection has been reported by numerous studies in experimental animal models [16,26–28]. After investigation, tested animals were euthanized by 100% CO_2_ inhalation for 5 min followed by cervical dislocation to minimize suffering. To perform virus challenge, mice were placed in an anesthetic inhalator chamber containing isoflurane (initial phase: 5%; maintenance phase: 1.5%–2.5%) for 1 min before s.c. or i.p. EV71 immunization.

### Cells, viruses, drugs, and antibodies

African green monkey kidney (Vero) (ATCC No. CCL-81) and human rhabdomyosarcoma (RD) (ATCC No. CCL-136) cells were provided by the Taiwan Centers of Disease Control (Taiwan CDC); the original cell lines were obtained from the American Type Culture Collection (ATCC), United States. Vero cells were cultured in a VP-SFM medium (Gibco-Invitrogen, CA, USA) supplemented with 4 mM L-glutamine (Gibco-Invitrogen, CA, USA). The RD cell line was cultured in DMEM medium containing 10% fetal bovine serum (Gibco-Invitrogen, CA, USA). Cells were maintained in a 37 °C incubator equilibrated with 5% CO_2_. Clinically isolated strains of EV71, E59 (B4) (GenBank: GQ150746.1), Neu (pinf7-54A) strain (C2) (GeneBank DQ060149), Tainan/5746/98 (C2) (GenBank: AF304457.1), and one strain of CVA16, 5079 (GenBank: AF177911.1) were obtained from Dr. Jen-Ren Wang, National Cheng-Kung University, Tainan, Taiwan, and were propagated in Vero cells based on the microcarrier cell culture bioprocess [29,30]. Human adenivirus 5 (Ad5; ATCC No. VR-1516) was purchased from ATCC and propagated in 293A cells. Virus stocks were stored at—80 °C. Virus stock titers were tested in a standard plaque-forming assay [31], and the number of plaque-forming units (pfu) was calculated.

The monoclonal antibody and Mab979 against the VP0/VP2 capsid protein of EV71 [32] was purchased from Millipore Inc. (MA, USA). A VP1-specific monoclonal antibody E1 was produced in-house, as described previously [32]. The anti-3C antibody was purchased from GeneTex (Cat. No. GTX630191). PE-Cy5-labeled mouse-specific CD4 and CD8 antibodies (Cat. No. 15-0042-82 and 15-0081-82, respectively) were purchased from eBioscience. PE-labeled rat anti-IFN-ɣ (Cat. No. 554412) was obtained from BD Bioscience. The anti-adenovirus type 5 antibody was obtained from Abcam (Cat. No. ab6982). Horseradish peroxidase (HRP)-conjugated donkey anti-mouse antibody (Cat. No. 715-036-150) and HRP-conjugated rabbit anti-goat antibody (Cat. No.305-035-003) were purchased from Jackson Immunoresearch Inc. (PA, USA).

### Ad-EVVLP and Ad-3CD construction and production

The *P1* and *3CD* genes of the EV71 Neu (pinf7-54A) strain were amplified by PCR and individually inserted into the shuttle vector pENTR4 (Invitrogen). The nucleotide element of the elongation factor-1α (EF-1α) promoter was inserted into the 3’ end of the *P1* gene and the 5’ end of the *3CD* insert to generate the pENTR4-P1/EF-1α/3CD construct. The *3CD* gene alone was inserted into pENTR4 to generate the pENTR4-3CD construct. The pENTR4-P1/EF-1α/3CD and pENTR4-3CD constructs were enzymatically recombined into the ΔE1/ΔE3 (replication-incompetent) Ad5 vector pAd/CMV/V5-DEST [33] to form recombinant pAd-EVVLP and pAd-3CD, respectively. pAd DNA was transfected into the 293A packaging cell line to produce the recombinant adenoviruses designated Ad-EVVLP and Ad-3CD. Ad-LacZ carrying a luciferase reporter gene as a vector control was obtained from Invitrogen. The recombinant viruses were purified and concentrated using Vivapure adenoPACK 100RT (Satorius Stedin Biotech). The purified virus titers were determined using a modified standard plaque assay. Various Ad virus dilutions were added to each well of 293A cells plated in a 6-well tissue culture plate. After overlaying the cultures with DMEM containing 0.75% methylcellulose, the cultures were incubated at 37 °C for 10 to 12 days and plaques were counted. The typical yield of adenoviruses was approximately 1 × 10^9^ pfu/mL.

### Western blot

Western blotting was performed as described previously [31]. Total cell lysates were prepared by treating 1 to 2 × 10^6^ cells with 100 μL ice cold lysis buffer (0.5% sodium deoxycholate, 0.1% sodium dodecyl sulfate (SDS), 0.5% NP-40, 50 mM TRIS, 150 mM NaCl) plus a protease inhibitor cocktail (Roche, French) and 1 mM PMSF (Sigma-Aldrich, CA, USA). Lysates were centrifuged for 20 min at 10,000 rpm at 4 °C to sediment the cell debris. The protein concentration of the cell lysates or fractions was measured using the Bradford method [34]. Cell lysates containing 10 μg protein were mixed with loading dye and loaded into each well of a 10% SDS-polyacrylamide gel (SDS-PAGE, Amersham Biosciences-GE Healthcare, USA) and subjected to electrophoresis in 1x Tris-glycine SDS-running buffer. The resolved proteins were transferred onto nitrocellulose membrane (Hybond-ECL, Amersham Biosciences-GE Healthcare, USA). Membranes were soaked in 5% skim milk in 1x PBS for 30 min at room temperature, then washed 3 times with 1x PBS plus 0.05% Tween 20 (PBS-T). The membrane was incubated with rat anti-3C (1:1000), MAB979 (1:5000), or anti-VP1 antibody (1:1000) for 14 to 16 h at 4 °C and subsequently washed with PBS-T followed by incubation with HRP conjugated anti-rat or donkey anti-mouse (for MAB979) antibodies. After 1 h incubation, the membrane was washed 5 times with PBS-T, and then Super Signal West Pico chemiluminescent substrate (Pierce, IL, USA) was layered onto the membrane, and it was exposed to X-ray film (Kodak, NY, USA). When necessary, the membranes were stripped using Restore buffer (Pierce, IL, USA) and blotted with another antibody.

### Flow cytometry

Splenocytes were harvested from BALB/c mice and labeled with 5-(6)-carboxyfluorescein diacetate succinimidyl ester (CFSE) (Cat. No. C34554, Molecular Probes). They were restimulated *in vitro* with 10^7^ pfu/mL UV-inactivated EV71 5746 or 1.4 μg/mL purified recombinant E59 3C proteins expressed by *E*. *Coli*. (provided by Dr. Pele Chong, a coauthor of this study) for 5 days. Proliferation of splenocytic CD4^+^ T cells was analyzed by flow cytometry (BD FACSCalibur) using PE-Cy5-labeled anti-CD4 antibodies. The population of no fluorescence signal-shifting in CFSE-prestained CD4^+^ T cells without antigen stimulation was set to 0%, and the population of negatively shifted CD4^+^ T cells (proliferating cells) after antigen stimulation was quantified. The mean percentage corresponding to the individually proliferating CD4^+^ T cells in each group was calculated. To detect the population of CD8^+^IFN-γ^+^ T cells, splenocytes were cocultured with the EV71 antigen for 2 days and then with brefeldin A (Cat. No. 00-4506-51, eBioscience) for 3 h before harvesting. Stimulated splenocytes were stained with PE-Cy5-labeled anti-CD8 antibody for 30 min, followed by subsequent fixation and permeabilization. A portion of these cells was further stained with PE-conjugated anti-IFN-γ^+^ antibody (BD Bioscience) for 30 min to detect intracellular IFN-γ. After washing, the samples were analyzed by flow cytometry.

### PCR and real time RT-PCR

pAd-EVVLP plasmid DNA was used as a template to detect the P1, 3CD, and EF-1α promoter regions within pAd-EVVLP by PCR using the respective primer pairs. The PCR conditions were as follows: 95 °C for 3 min; 35 cycles at 95 °C for 1 min, 60 °C for 1 min, and 72 °C for 3 min; and a final incubation at 72 °C for 2 min.

Total RNA was purified from tissues using TRIZOL reagent (Invitrogen, CA, USA) following the manufacturer’s instructions and was subjected to real time RT-PCR. Total RNA was converted into cDNA using random primers (Genomics BioSci&Tech, Taiwan) and reverse transcriptase (Bionovas, Toronto, Canada). The synthesized cDNA was subjected to quantitative PCR analysis (LightCycler 480 SYBR Green Real-Time PCR system) using primer pairs specific to the VP1 region of EV71 P1 RNA. Human β-actin gene expression was used as an internal control. The PCR conditions were as follows: 95 °C for 3 min; 40 cycles at 95 °C for 10 s, 65 °C for 20 s, and 72 °C for 2 s; and a final incubation at 72 °C for 2 min. The number of cycles required for amplification of transcripts was obtained. The relative expression of EV71 P1 RNA was calculated as follows: the individual Ct obtained from the experimental group or control group was subtracted by its respective Ct (β-actin) to gain normalized Ct. Then, 2^Normalized Ct (VP1 of P1 RNA from the sample without viral infection)^ was divided by 2^Normalized Ct (VP1 of P1 RNA from the sample with viral infection)^. The forward and reverse primers, [5_-ACGCGCAAATGCGTAGAAAGGT-3_—forward and 5_-TTAGTGGCAGTTTGCCATGCGA-3_—reverse], were used to amplify and detect VP1 RNA. human β-actin mRNA was amplified using the primer pairs 5_-ACCAACTGGGACGACATGGAGAAA-3_—forward and 5_-TAGCACAGCCTGGATAGCAACGTA-3_—reverse. Primer pairs targeting the P1, 3CD, and EF-1α promoter regions of Ad-EVVLP are as follows: P1: 5_-ATCG GAATTCATGGGCTCACAGGTGTCCAC-3_—forward and 5_-CTTGTCGACTTAGAGAG TGGTAATTGCTG-3_—reverse, 3CD: 5_-ATCGGAATTCATGGGGCCGAGCTTGGAC-3_—forward and 5_-ATCGCTCGAGAAACAATTCGAGCC-3_—reverse, EF-1α promoter: 5_-ATCGACGCGTGTGAGGCTCCGGTGCCC-3_—forward and 5_-ATCGCCCGGGGTTTTCACGACACCTG-3_—reverse. All primer sets were commercially synthesized by Genomics BioSci&Tech, Taiwan.

### Density gradient purification of EV71 VLP and Ad

HEK-293A cells (1 x 10^7^) were seeded in a 10-well plate 1 day prior to Ad-EVVLP infection with MOI = 1. After 24 h of infection, the cells were harvested and lysed in 1% NP-40 lysis buffer (50 mM Tris/HCl, pH 7.5, 150 mM NaCl, 2 mM EDTA, and 1% NP-40) on ice for 30 min and centrifuged at 1000 xg for 10 min to remove the cell debris. The supernatants were harvested and concentrated by ultracentrifugation at 100,000 xg for 1 h at 4 °C and then dissolved in 30 μL PBS. The samples were loaded into self-generated iodixanol gradients, which were prepared by mixing 0.6 mL solution S (0.25 M sucrose, 15 mL EDTA, 30 mM Tris/HCl, pH 8.0) and 0.42 mL 60% (w/v) iodixanol (Cat. No. 1114542, Optiprep; Axis Shield, UK) to form a homogenous solution. Gradients were generated through centrifugation at 162,000 xg for 24 h at 4 °C. The various fractions were manually harvested from the top (named Fraction No. 1), 0.1 mL per fraction, and 10 fractions were serially collected for each sample. These fractions were subjected to Western blot using Mab979 antibodies or transmission electron microscopy.

### Transmission electron microscopy

HEK-293A cells were harvested 24 h after Ad-EVVLP infection, and cell pellets were frozen and thawed twice at—80 °C for 30 min and 37 °C for 15 min. Lysates were centrifuged at 3000 rpm for 15 min at room temperature, and supernatants were harvested and subjected to examine adenovirus using a JEOL JEM-1400 transmission electron microscope (TEM). The lysate was fractionated through density gradient centrifugation, and fractions were concentrated through ultracentrifugation at 100,000 xg for 1 h and resuspended in 200 μL PBS. The fractions were then cleaned by centrifugation in a 100 KDa-cut-off spin-X^R^ UF 20 column (Corning). Samples were treated with uranyl acetate and inspected by TEM.

### ELISA

To detect anti-EV71, anti-Ad, or anti-3C antibodies in sera, 96-well plates were coated with 100 μL per well of heat-inactivated (56 °C for 1 h) 10^3^ pfu EV71 5746 (C2 genotype) or E59 (B4 genotype) strains, 200 pfu purified Ad5, or 700 ng recombinant 3C protein in carbonate coating buffer. Serum samples collected from immunized mice were inactivated at 56 °C for 30 min. Two-fold serial dilutions of the sera were performed beginning from an 8-fold initial dilution. The diluted sera were added to the wells and incubated at room temperature for 2 h. After washing with PBS-T, HRP-conjugated donkey anti-mouse IgG antibodies were added to the wells for 45 min. The reaction was developed by incubation with 100 μL TMB substrate (3, 3’, 5, 5’-etramethyllbenzidine) for 20 min in the dark and terminated by adding 50 μL 2 N H_2_SO_4_. The optical density at 450 nm was determined using a microplate absorbance reader (SPECTRA, MAX2, M2). To detect cytokines secreted by splenocytes, the supernatants from 2-day cultures of splenocytes restimulated with 10^7^ pfu/mL UV-inactivated EV71 5746 were analyzed using a calorimetric sandwich IFN-γ, IL-4, IL-13, and IL17A ELISA kit (Cat. No. 887314, 88–7044, 88–7137, and 88–7371, respectively, eBioscience). The assays were conducted according to the manufacturer’s instructions, and the optical densities at 450 nm were determined using a microplate absorbance reader.

### Neutralizing assay

To detect the neutralizing activity as described in our previous study [32], each sample was serially diluted 2-fold in fresh cell culture medium. A total of 100 μL 100 TCID_50_ virus suspension, E59, 5746, or CVA16 strain was added to each tube containing 100 μL serially diluted serum. After incubation at 4 °C for 18 to 24 h, 100 μL virus serum mixture was added to 96-well plates seeded with rhadomyosarcoma (RD) cells and incubated for 7 days at 37 °C; TCID_50_ values were measured by counting cytopathic effects (CPE). The 50% neutralization inhibition dose (ID50) was calculated as the reciprocal of the serum dilution compared to normal serum using the Reed–Muench method [35]. A mouse anti-EV71 Mab979 antibody (Chemicon International) was used as an internal positive control.

### Enzyme-linked immunosorbent spot assay

Suspensions containing 5 × 10^6^ RBC-free splenocytes were prepared from individual mice and seeded in individual wells of 96-well filtration plates (Millipore) pre-coated with capturing monoclonal antibodies for murine IL-4 or IFN-γ (0.5 μg/well) (Cat. No. 16-7041-68 or 16-7313-68, respectively, eBioscience) and blocked with conditioned medium (CM) for 1 h at room temperature. The splenocytes were added to 10^6^ pfu/well UV-inactivated EV71 5746 dissolved in CM (100 μL). Splenocytes incubated with Con A (10 g/mL) were used as a positive control. Unstimulated splenocytes were used as a negative control. Plates were maintained in a 37 °C incubator equilibrated with 5% CO_2_ for 48 h. The individual wells of the ELISPOT plates were washed 3 times with PBS-T, and 0.2 g of the corresponding biotinylated detection monoclonal IL-4- or IFN-γ-specific antibody was added to detect the respective cytokines. The plates were washed after 2 h incubation at room temperature, and 100 L streptavidin-alkaline phosphatase (1:250 dilution) was added to the individual wells. The plates were incubated at room temperature for 45 min. Finally, the plates were washed 4 times with wash buffer, and 100 L AEC (3-amine-9-ethylcarbazole, Sigma-Aldrich) substrate was added to each well and allowed to react for 30 min at room temperature in the dark. The plates were washed with water, air-dried overnight, and the spots on each well were scored using an immunospot counting reader (Immunospot, Cellular Technology Ltd.). The results were expressed as the number of cytokine-secreting cells per 5 × 10^5^ splenocytes seeded in the initial culture.

### Ad-EVVLP vaccination of hSCARB2 transgenic mice challenged with EV71 and CVA16

hSCARB2-Tg mice in a C57BL/6 background generated were previously generated by our group and were maintained by cross-mating hSCARB2-Tg subjects to obtain inbred mice [16]. One-day-old hSCARB2-Tg mice were inoculated s.c. with PBS, 3 × 10^7^ pfu Ad-LacZ, or 3 × 10^6^ or 3 × 10^7^ pfu Ad-EVVLP, or 1 μg FI-EV71 vaccine on Days 1 and 7, and then challenged s.c. with 3 × 10^6^ pfu EV71 5746 or 5 × 10^5^ pfu CVA16 on Day 14. The mice were monitored daily for survival for 15 days after the challenge.

### Accession numbers

The list of accession number/ID numbers for cells and viruses was shown below:

ATCC No. CCL-81: African green monkey kidney (Vero) cell. ATCC No. CCL-136: Human rhabdomyosarcoma (RD) cell. GenBank, GQ150746.1: EV71, E59 (B4). GeneBank, DQ060149: EV71 Neu *(*pinf7-54A) (C2). GenBank, AF304457.1: EV71 Tainan/5746/98 (C2). GenBank, AF177911.1: CVA16, 5079. ATCC No. VR-1516: Human adenovirus type 5

## Results

### Ad-EVVLP and VLP production in HEK-293A cells

**Fig 1:**
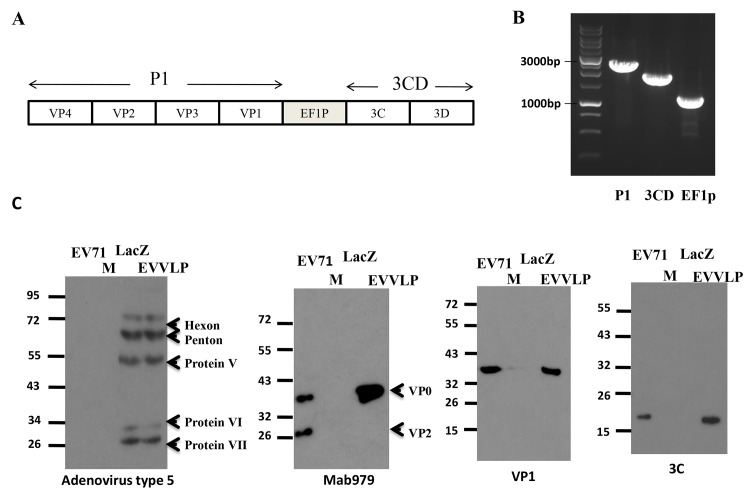
Construction of a recombinant adenovirus vector carrying EV71 P1 and 3CD genes and expressing VLPs. (A) Adenovirus construct; Ad-EVVLP expresses the P1 gene, which is comprised of VP1 to VP4 subunit sequences driven by the CMV promoter and the 3CD gene driven by elongation factor-1α promoter (EF1p). (B) Specific primers against the P1, 3CD, and EF1p sequences were used to amplify and detect the insertion of P1, 3CD, and the EF-1α promoter in the Ad-EVVLP construct. (C) Ad-LacZ and Ad-EVVLP produced from the lysates of 293A transfectants were analyzed by immunoblotting with the polyclonal anti-Ad5 antibody. The cascade of VLP formation was shown; the translated P1 polypeptide was produced and cleaved by the 3CD protease, which was expressed from 3CD RNA to obtain the individual VP0, VP1, and VP3 subunit proteins. VP0, VP1, and 3C were detected by blotting with the Mab979 monoclonal antibody, or VP0, VP1, or 3C-specific antibodies. A total of 10^3^ pfu per well of purified EV71 5746 as antigens for immunoblotting control was included. The lane containing the protein marker (M) is marked.

We used the E1- and E3-deleted adenovirus-5 genome to construct the Ad-EVVLP expression vector, which carried full-length *P1* and *3CD* genes of EV71 (Fig 1A). We performed polymerase chain reaction (PCR) to confirm the inserts: *P1*, *3CD*, and elongation factor-1α promoters (EF-1p) using the respective primers in the Ad-EVVLP construct. The PCR products corresponded to 2585 bps (*P1*), 2020 bps (*3CD*), and 1186 bps (EF-1p) (Fig 1B). Upon transfection in competent HEK-293A cells constitutively expressing the E1 protein, the recombinant Ad was generated. We examined Ad-EVVLP and the control Ad-LacZ by western blot using the polyclonal anti-Ad5 antibody. They each expressed Ad structural proteins, including hexon, penton, and protein V, VI, and VII (Fig 1C). Previous studies have shown that multiple capsid proteins of VP0 (38 KDa, a precursor product of VP2+VP4), VP1 (36 KDa), VP2 (28 KDa), VP3 (25 KDa), and VP4 (8 KDa) can be detected in EV71-infected cells [32,36]. The VLP expression by Ad-EVVLP was characterized; translated products of EV71 VLPs, including VP0 (a precursor of VP4-VP2) but not VP2, were detected in 293A cells using the VP2-specific monoclonal antibody Mab979. EV71 antigens VP0 and VP2 were detected by Western blotting (Fig 1C). We confirmed VP1 expression using a VP1-specific antibody, which corresponded to the 34 to 36 kDa bands in Ad-EVVLP-infected lysates and in the sample of EV71 antigens (Fig 1C). The antigenic profile of VLPs expressed by Ad-EVVLP was similar to E-particles (composed by VP0, VP1, and VP3) from EV71, which does not contain viral RNA, compared to F-particles (composed by VP2, VP4, VP1, and VP3). In addition, 3C’ processed the P1 polyprotein to form VP0, VP1, and VP3 in the absence of EV71 genetic RNA [36]. We detected an 18 kDa band using an anti-3C antibody. In contrast, we could not detect these bands in uninfected or Ad-LacZ-transfected 293A cells (Fig 1C). However, no VP3-specific antibody is available to detect VP3. We could not detect a VP3 signal by blotting with sera from EV71-infected mouse (Fig A in S1 Text).

**Fig 2:**
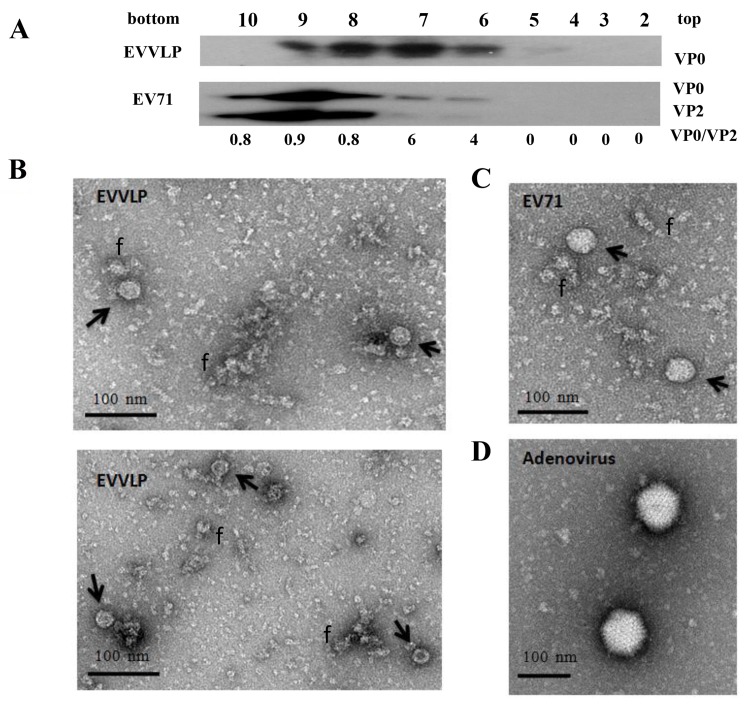
Coproduction of VLPs and Ad-EVVLP particles in Ad-infected cells. (A) The 293A cells were infected with MOI = 0.1 Ad-EVVLP. Cell lysates were prepared 24 h post-infection and fractionated in iodixanol density-gradients as described in the Materials and Methods. Fractionation of 10^7^ pfu EV71 5746 strain produced from Vero cells. Each fraction was subjected to Western blot using the Mab979 antibody. Signal intensity of VP0 and VP2 bands were quantified by Image-Pro Plus 6.0 software, and the ratio of VP0/VP2 was calculated and shown below. (B) The pooled Fractions 7 and 8 of Ad-EVLP and (C) Fractions 8 and 9 of EV71 samples were treated with uranyl acetate for inspection by TEM. Arrows indicate the complete particles corresponding to the 30-nm-diameter VLPs. The fractured VLPs marked as f are present. (D) Approximately 100-nm-diameter adenovirus particles from the lysate of Ad-EVVLP-infected cells without fractionation were also inspected by TEM and shown.

To demonstrate that the EV71 VLP particles were cogenerated in Ad-producing cells, we purified the virions from the cytosol of Ad-EVVLP transfectant using fractionation through density gradient centrifugation. We fractionated EV71 particles as a control. We characterized each fractionated sample by Western blot analysis using Mab979. The major band was 38 kDa, corresponding to VP0, but there was minor expression of a 28 kDa corresponding to VP2 (intensity ratio of VP0/VP2 = 4 and 6, respectively), which together make the E-particle of EV71 in Fractions 6 and 7. In contrast, the opposite pattern of VP0/VP2 expression (0.8, 0.9, and 0.8, respectively) corresponding to F-particles was observed in Fractions 8, 9, and 10. However, only VP0 signals were detected in Fractions 6 to 9 of Ad-EVVLP-infected lysate (Fig 2A). A similar antigenic profile of EV71 has been previously reported [37].

We pooled Fractions 7 and 8 of Ad-EVVLP samples and examined them by TEM. TEM analysis revealed some fractured VLPs (f) and cellular impurities in the samples due to sample preparation. Two sizes of complete particles were also present; particles over 100 nm in diameter corresponded to Ad particles (Fig 2D), and particles approximately 30 nm in diameter corresponded to VLPs expressed by Ad-EVVLP (Fig 2B). EV71 particles in the pool of Fractions 8 and 9 of EV71 sampleswere also examined (Fig 2C).

### EV71 VLP-specific humoral responses in Ad-EVVLP-immunized mice

**Fig 3:**
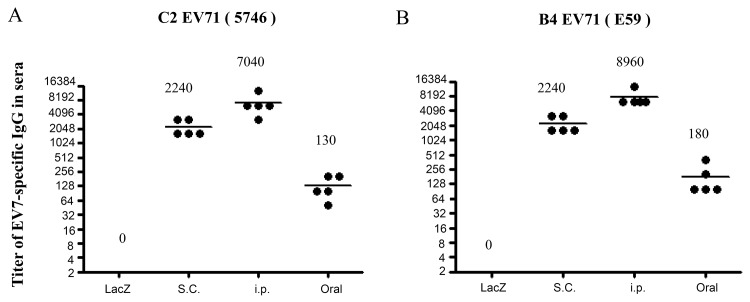
Ad-EVVLP induction of EV71-specific IgG cross-reacts E59 and 5746 strains. Seven-week-old BALB/c mice were individually primed and boosted at 14-day intervals though oral, s.c., or i.p. routes with 10^8^ pfu Ad-EVVLP or Ad-LacZ. Serum samples collected on Day 21 were assayed for IgG against heat-inactivated (A) 5746 and (B) E59-immobilized ELISA. The results were expressed as titers for each test sample. Bars correspond to mean titers for each experimental group of 5 mice.

To examine the immunogenicity of Ad-EVVLP compared to the FI-EV71 vaccine, we intraperitoneally (i.p.), subcutaneously (s.c.), or orally administered adult BALB/c mice with 1 × 10^8^ pfu of Ad-EVVLP or Ad-LacZ on Days 1 and 14. Animals in separate groups were s.c. administered 0.1 μg or 1 μg FI-EV71 twice to evaluate the virus-specific immune responses compared with those of recombinant adenoviruses. The results of ELISA assays showed (Fig 3A) that the mean anti-EV71 titer against EV71 5746 (C2 subgenotype) in Ad-EVVLP-immunized serum samples collected on Day-21 were 2240, 7040, and 130 for s.c., i.p., and orally, respectively. We did not detect a titer in serum from s.c. Ad-LacZ-immunized mice (Fig 3A). The mean titer of serum antibodies reacting with the EV71 E59 strain (B4 subgenotype) from the Ad-EVVLP-immunized animals was to 2240, 8960, and 180, for s.c., i.p., or orally, respectively. Again, no E59 reactivity was detected in serum of the mice immunized with Ad-LacZ (Fig 3B). Sera from Ad-EVVLP-immunized mice possessed EV71 neutralizing activity (Table 1). Higher virus neutralization titers (1/128) were found in i.p. and s.c. Ad-EVVLP-immunized mice compared to a considerably low neutralizing titer in orally administered animals. Neutralizing antibodies produced in Ad-EVVLP-immunized mice exhibited potent neutralizing activity against EV71 B and C strains. Comparable titers (1/256 and 1/512) of neutralizing antibody in the mice s.c. administered 0.1 μg FI-EV71 vaccine. No anti-CVA16 neutralizing activity was found in the serum from mice immunized with Ad-EVVLP, FI-EV71 vaccine, or PBS (< 1:8; Table 1). These results are consistent with previous reports [38] that FI-EV71 vaccine could not elicit cross-neutralizing antibody against CVA16.

**Table 1:** Induction of neutralizing antibodies against EV71 E59 and 5746 strains and CVA16 by Ad-EVVLP or FI-EV71 vaccine.

	Route	Strain	EV71-specific neutralizing antibody titers (mean)
Ad-LacZ	i.p/s.c/oral	E59/5746/CVA16	<1:8
Ad-EVVLP	i.p.	E59	1:64
		5746	1:128
		CVA16	<1:8
	s.c.	E59	1:64
		5746	1:64
		CVA16	<1:8
	oral	E59	<1:8
		5746	<1:8
FI-EV71 (0.1 μg)	s.c.	E59	1:256
FI-EV71 (1 μg)	s.c.	5746	1:512
		CVA16	<1:8

Seven-week-old BALB/c mice were individually primed and boosted at 14-day intervals i.p., s.c., or orally with 10^8^ pfu Ad-EVVLP or Ad-LacZ. Sera collected on Day 21 were analyzed for neutralizing activity by incubating 10^2^ pfu 5746, E59 or CVA16 with varying dilutions of individual immune sera before being added to RD cells. CPE was observed after 5 days of culture. The results were expressed as neutralizing titers that correspond to the dilution of immune sera, giving TCID_50_ value of 50% reduction of cytopathic effect.

### Induction of VLP-specific cellular immunities in Ad-EVVLP-immunized mice

**Fig 4:**
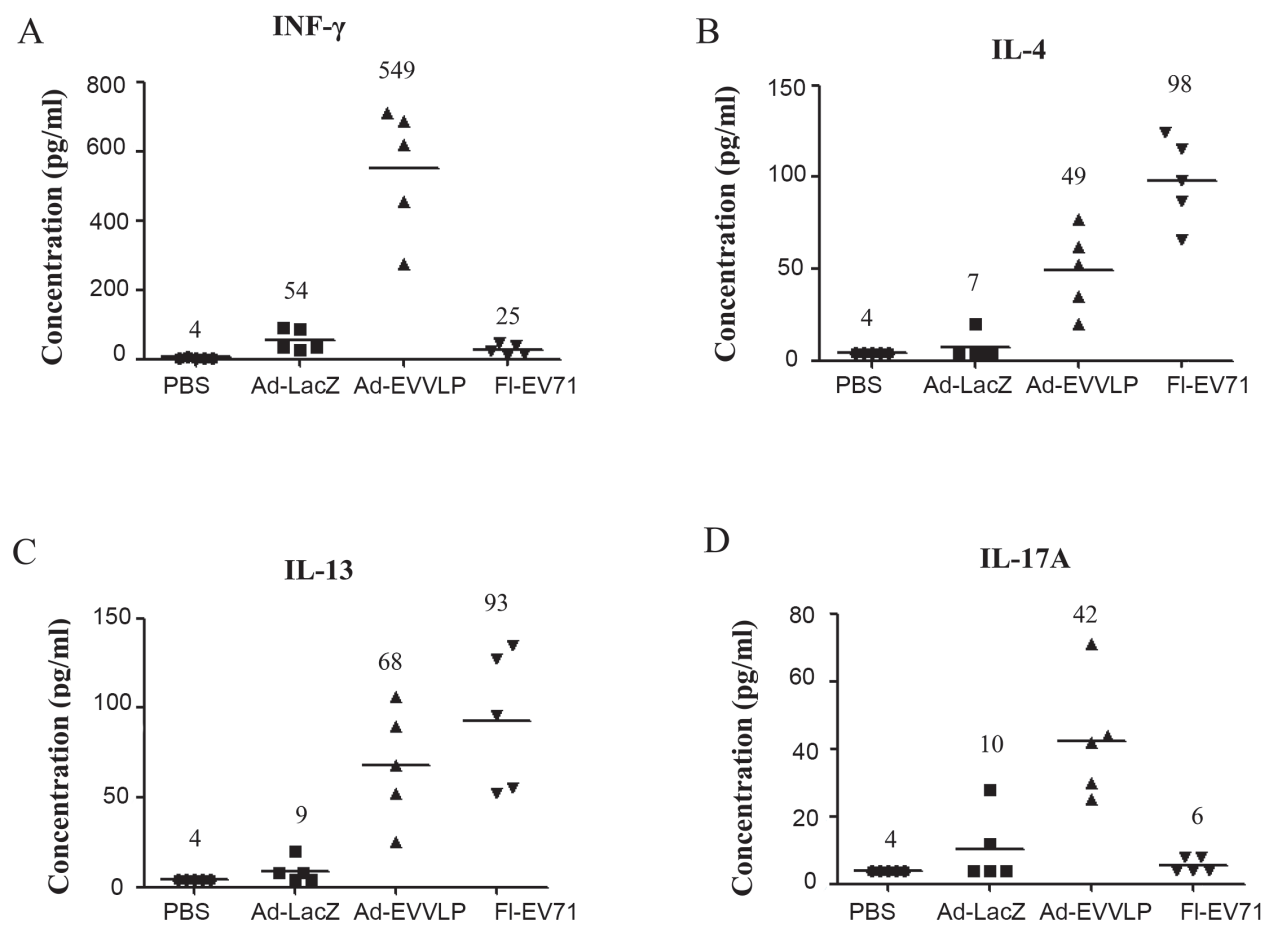
Induction of VLP-specific cytokine responses in Ad-EVVLP-immunized mice. (A) Mice immunized twice s.c. with PBS, Ad-LacZ, Ad-EVVLP, or FI-EV71 were sacrificed on Day 7 after vaccine boost. Splenocytes were collected and cultured in the presence or absence of 10^7^ pfu/mL UV-EV71 5746 for 48 h. Culture medium was collected and quantitated for (A) IFN-γ, (B) IL-4, (C) IL-13, and (D) IL-17A by ELISA using the protocols described in the Materials and Methods section. Five mice per group were tested. The results were presented as the cytokine concentration in picograms per milliliter.

Recent studies on host immune responses against EV71 have suggested that T cell immunity plays a critical role in the protection against EV71 infection and control of the disease [39,40]. Therefore, we investigated whether the VLP-specific CD4^+^ and CD8^+^ T cell responses could be elicited in Ad-EVVLP-immunized mice. Seven days post-immunization, we isolated lymphocytes from the spleen, followed by *in vitro* restimulation with UV-inactivated EV71 (UV-EV71). Lymphocytes from Ad-LacZ-immunized mice produced background IFN-γ levels. In contrast, substantially higher IFN-γ levels were measured in lymphocyte cultures from mice administered Ad-EVVLP (Fig 4A). Lymphocytes from FI-EV71 vaccine-immunized mice secreted background IFN-γ levels (Fig 4A). Within the panel of Th2 cytokines assayed, IL-4 (Fig 4B) and IL-13 (Fig 4C) were moderately secreted by lymphocytes from Ad-EVVLP-immunized mice, indicating that balanced Th1/Th2 responses were activated. Interestingly, immunization of the FI-EV71 vaccine led to the production of the highest IL-4 and IL-13 levels, indicating that a Th2 biased response was induced (Fig 4B and 4C). This result supports our findings that FI-EV71 vaccination in hSCARB2-Tg mice induced splenocytic IL-4 but not IFN-γ secretion, as shown previously [16]. The results obtained from IFN-γ and IL-4 ELISPOT assays confirmed that i.p. Ad-EVVLP immunization induced significant splenocytic IFN-γ production and low levels of IL-4 secretion in Ad-EVVLP-vaccinated mice (Fig B in S1 Text). A considerable amount of IL-17A was produced by splenocytes from Ad-EVVLP-immunized mice in response to EV71 antigens. This was in sharp contrast to the barely detectable amount of IL-17 secreted by splenocytes of animals immunized with Ad-LacZ or the FI-EV71 vaccine (Fig 4D). These results indicate that Ad-EVVLP drives T cell activation, leading to the differentiation of a subpopulation of T cells that bear the Th1, Th2, and IL-17 producing phenotypes.

**Fig 5:**
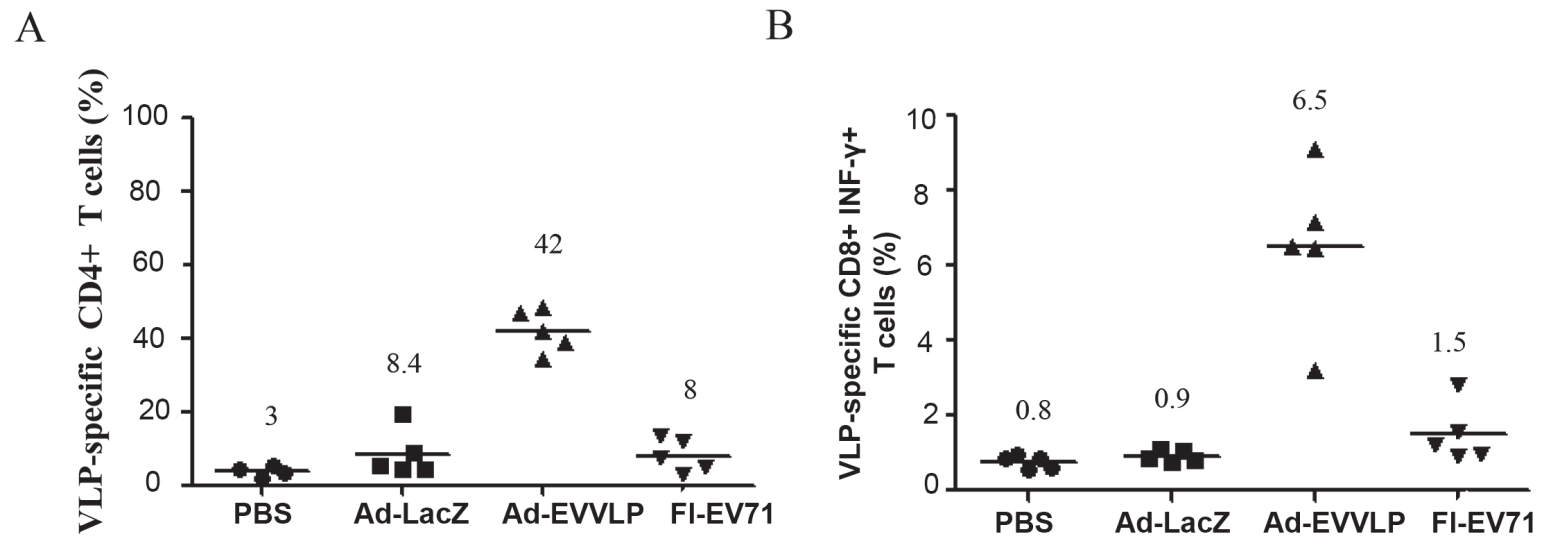
Induction of VLP-specific CD4^+^ and CD8^+^ T-cell responses in Ad-EVVLP-immunized mice. Splenocytes from individual mice immunized twice s.c. with PBS, Ad-LacZ, Ad-EVVLP, or FI-EV71 were cultured in the presence or absence of 10^7^ pfu UV-EV71 5746 for 48 h. (A) CD4^+^ T cell proliferation in response to EV71 particles was analyzed using flow cytometry with PE-Cy5-conjugated anti-CD4 antibodies. (B) Splenocytes were stained with PE-Cy5-conjugated anti-CD8 antibodies and subsequently fixed and stained with PE-conjugated anti-IFN-ɣ antibodies, and analyzed using flow cytometry. The results were presented as the mean of the percentage of CD4^+^ or CD8^+^ T cells after antigen stimulation compared to the gated CD4^+^ T cells without antigen stimulation that were set as 0%. Five mice per group were assayed.

We measured VLP-specific CD4^+^ T cell proliferation in vaccine-immunized splenocytes followed by restimulation with UV-EV71 by examining the negative shift of fluorescent signal in 5-(6)-carboxyfluorescein diacetate succinimidyl ester (CFSE)-prestained CD4^+^ T cells using flow cytometry. Compared to little or no shift of signals in the PBS- and Ad-LacZ-immunized groups (3% and 8.4%, respectively), a substantial shift was detected in Ad-EVVLP-immunized group (42%; Fig 5A). The proliferation of CD4^+^ T cells corresponding to UV-EV71 was barely detectable in the FI-EV71-immunized mice (8%; Fig 5A), indicating that the antigenicity of FI-EV71 reacting to VLP was altered, and therefore the immunized CD4^+^ T cells could not be fully reactivated by exposure to EV71 particles. We further examined the response of VLP-specific CD8^+^ T cell activation in Ad- and FI-EV71-vaccinated animals. After UV-EV71 restimulation, we stained splenocytes with fluorescence dye-conjugated antibodies reacting to surface CD8 molecules and intracellular IFN-γ and analyzed the cells by flow cytometry. We found that the number of CD8^+^IFN-γ^+^ T cells in Ad-EVVLP-immunized mice (6.5%) was higher than in Ad-LacZ- or FI-EV71-immunized mice (0.9% or 1.5%, respectively; Fig 5B). These results suggest that Ad-EVVLP activates EV71 VLP-specific cellular immunity.

### Ad-EVVLP vaccine confers protection against EV71 infection in hSCARB2-Tg mice

**Fig 6:**
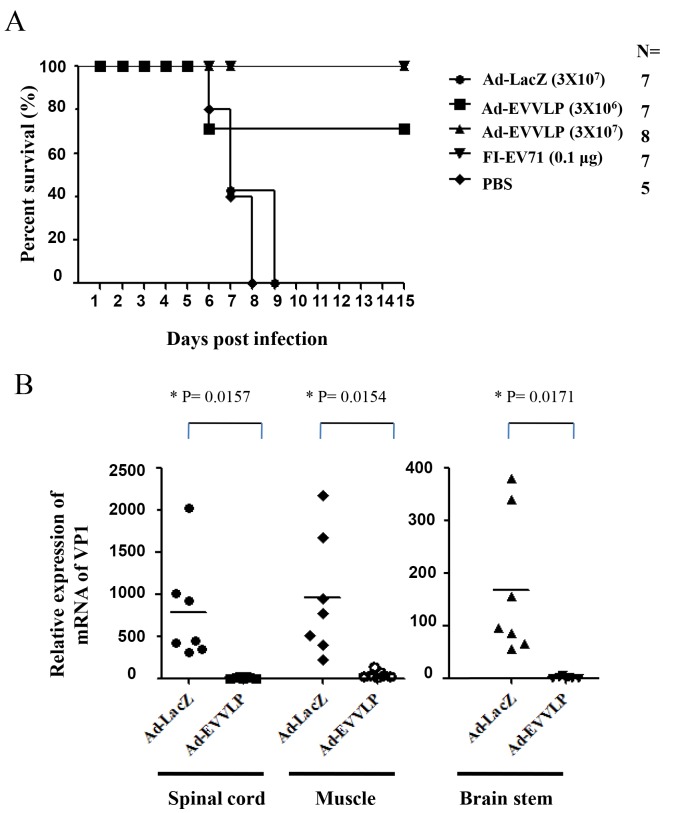
Ad-EVVLP confers protection against EV71 in hSCARB2-Tg mice. (A) Survival of hSCARB2-Tg mice pre-immunized twice s.c. with PBS (♦) 3 × 10^7^ pfu Ad-LacZ (●), or 3 × 10^6^ (■) or 3 × 10^7^ (▲) pfu Ad-EVVLP, or 0.1 μg FI-EV71 vaccine (▼) on Days 1 and 7 after birth prior to being s.c.challenged with 3 × 10^6^ pfu EV71 5746. The number (N) of transgenic mice is shown in the figure. A log-rank test was used for statistical analysis. (B) On Day 4 post-infection, mice were sacrificed and RNAs were extracted from the brainstem, spinal cord, and muscle for quantitative RT-PCR, using primers specific to the VP1 region of EV71 RNA. Quantitative RT-PCR using primers specific to the β-Actin gene was used as the internal control. Relative VP1 mRNA expression in the individual Ad-EVVLP-vaccinated tissues was normalized to β-actin expression in each individual sample and then to the mean of relative normalized VP1 mRNA expression in Ad-LacZ-vaccinated samples. The mean relative expression in each group of 7 mice was calculated. Unpaired student’s *t*-tests with Welch corrections were used for statistical analysis.

We further assessed the efficacy of Ad-EVVLP in protecting against EV71 infection using the hSCARB2-Tg mice model. One-day-old hSCARB2-Tg mice were primed and s.c. boosted with Ad or FI-EV71 vaccine on Days 1 and 7, followed by s.c. challenge of 3 × 10^6^ pfu EV71 5746 strain 14 days after birth. Mice were monitored daily for survival. As shown in Fig 6A, mice immunized with 3 × 10^7^ pfu Ad-LacZ or PBS died 8 to 9 days after challenge. In contrast, 75% of the mice survived after receiving as little as 3 × 10^6^ pfu of Ad-EVVLP, and 100% of the mice survived when injected with a 10-fold higher dose of Ad-EVVLP. In comparison, EV71-challenged mice received 0.1 μg FI-EV71 vaccine and 100% of the mice survived (Fig 6A), indicating that the protective efficacy of Ad-EVVLP against EV71 infection was comparable to the FI-EV71 vaccine. We further examined the viral loads in different tissues of vaccine-immunized animals followed by viral challenge. We extracted RNA from various organs of EV71-challenged Tg mice on Day 4 post-infection to quantify EV71 transcripts using real time RT-PCR with VP1 region-specific primers. Ad-EVVLP immunization substantially reduced VP1 expression in the brainstem, spinal cord, and muscle, compared to considerably high expression in Ad-LacZ-vaccinated mice (Fig 6B), confirming that Ad-EVVLP can suppress EV71 infection and replication.

### 3C-specific immune responses generated in Ad-EVVLP-immunized mice

**Fig 7:**
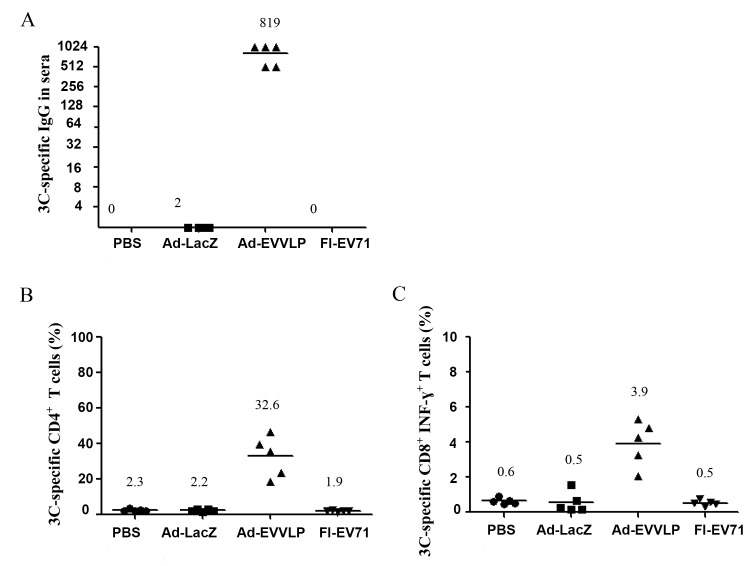
Induction of 3C-specific antibody and CD4^+^ and CD8^+^ T-cell responses in Ad-EVVLP-immunized mice. BALB/c mice were individually primed and boosted at an interval of 14 days s.c. with PBS, 10^8^ pfu Ad-EVVLP or Ad-LacZ, or 0.1 μg FI-EV71. Mice were sacrificed, and serum and splenocytes were collected on Day 21. (A) Serum was assayed for IgG against recombinant 3C-immobilized ELISA. The results are expressed as titers for each test sample. Bars correspond to the mean titers for each experimental group. Splenocytes were cultured in the presence or absence of 1.4 μg recombinant 3C protein for 48 h. (B) The proliferation of CD4^+^ T cells in response to 3C was analyzed by flow cytometry with PE-Cy5 antibodies against CD4. (C) Activated CD8^+^ T cells in splenocytes were stained with PE-Cy5-conjugated anti-CD8 antibodies and subsequently fixed and stained with PE-conjugated anti-IFN-ɣ antibody, and then analyzed using flow cytometry. The results were presented as the mean percentage of CD4^+^ or CD8^+^ T cells after antigen stimulation, compared to gated CD4^+^ T cells without antigen stimulation that was set at 0%. Five mice per group were assayed.

3C and 3D are proteins conserved between EV71 and CVs (A16, A6, A10, and A4) that share at least 90% homology in their amino acid sequences (Table A in S1 Text). Technical limitations restricted our ability to obtain recombinant 3D protein, but we were able to generate recombinant 3C protein. We therefore examined whether 3C-specific immunities were induced by Ad-EVVLP vaccination. We collected and assayed serum from mice on Day 7 post-prime-boost s.c. with Ad-EVVLP, Ad-LacZ, or FI-EV71. Serum from Ad-EVVLP-immunized mice elicited activity against 3C protein in a recombinant 3C-protein-coated ELISA capturing assay. Anti-3C binding activity was not detected in serum from Ad-LacZ- or FI-EV71-immunized mice (Fig 7A). Like antisera obtained from FI-EV71, antisera from Ad-EVVLP-immunized mice showed no virus neutralizing activity against CVA16 (Table 1). Moreover, serum from mice primed with 10 μg recombinant 3C protein formulated with complete Freund’s adjuvant (CFA) and boosted with the same dose of 3C protein adjuvanted with incomplete FA (IFA) at an interval of 14 days elicited 3C-binding activity, but did not neutralize EV71 or CVA16 infection (Fig C in S1 Text). Taken together, these results suggest that the induction of 3C-specific antibody does not contribute to the protection against EV71 infections.

We further examined 3C-specific cellular immunity in mice immunized with Ad-EVVLP. We isolated splenocytes on Day 7 after vaccine boost and restimulated them with recombinant 3C protein *in vitro* and observed of CD4^+^ and CD8^+^ T cell activation by flow cytometry (Fig 7). CD4^+^ T cells from the Ad-EVVLP-immunized group responding to 3C were activated (mean = 32.6%), but there were no or minimally activated splenocytes in the PBS-, Ad-LacZ-, and FI-EV71-immunized mice (mean = 2.3%, 2.2%, and 1.9%, respectively; Fig 7B). Activated CD8^+^ (CD8^+^IFN-γ^+^) T cells corresponding to 3C protein in the Ad-EVVLP-immunized splenocytes were markedly activated (mean = 3.8%), in contrast to the minimal CD8^+^IFN-γ^+^ T cells in Ad-LacZ (mean = 0.5%) or FI-EV71 (mean = 0.5%) and background levels of CD8^+^IFN-γ^+^ T cells obtained from mice immunized with PBS buffer alone (mean = 0.6%; Fig 7C). These results confirm that Ad-EVVLP can induce CD4^+^/CD8^+^ T cell responses against VLP and 3C protein.

### Ad-EVVLP vaccine confers protection against CVA16 infection in hSCARB2-Tg mice

**Fig 8:**
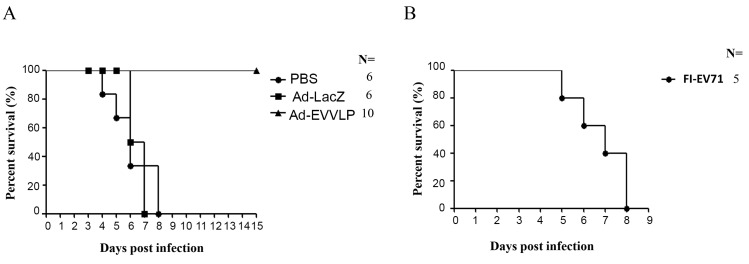
Ad-EVVLP but not FI-EV71 protects hSCARB2-Tg mice from CVA16 challenge. One-day-old hSCARB2-Tg mice were pre-immunized twice s.c. with (A) PBS (●), 3 × 10^7^ pfu Ad-LacZ (■), or 3 × 10^7^ (▲) pfu Ad-EVVLP, or (B) 1 μg FI-EV71 vaccine (●) on Days 1 and 7 after birth prior to being challenged s.c. with 5 × 10^5^ pfu CVA16. The survival of mice was monitored on a daily basis for 15 days. The number (N) of transgenic mice was shown. A log-rank test was used for statistical analysis.

In addition to the protection against EV71 infection, we investigated whether Ad-EVVLP or FI-EV71 can facilitate hSCARB2-Tg mice in resisting lethal CVA16 challenge. After Ad-EVVLP immunization, 100% of hSCARB2-Tg mice survived, in contrast to 0% survival of hSCARB2-Tg mice that received PBS or Ad-LacZ after CVA16 challenge (Fig 8A and Table 2). Ad-EVVLP fully protected animals challenged with a 6-fold higher CVA16 dose (3 × 10^6^ pfu; Table 2). Immunization with 1 μg FI-EV71 vaccine did not protect hSCARB2-Tg mice against 5x10^5^ pfu CVA16 challenge, leading to 0% survival (Fig 8B and Table 2). Taken together, these results suggest that the Ad-EVVLP vaccine elicits potent CD4^+^/CD8^+^ T cell immune responses to control EV71 and CVA16, whereas the FI-EV71 vaccine protects against only EV71 challenge. This demonstrated a correlation with the results shown in Table 1, and the results of phase I clinical trials in which sera from subjects immunized with FI-EV71 vaccine neutralized distinct EV71 genotypes, but could not cross-neutralize CV [21,38].

**Table 2:** Ad-EVVLP protects hSCARB2-Tg mice from CVA16 challenge.

	CVA16 (pfu)	Survival (%)
**PBS**	5 x 10^5^	0 (6/6)*
**Ad-LacZ**	5 x 10^5^	0 (6/6)
**Ad-EVVLP**	5 x 10^5^	100 (10/10)
	3x 10^6^	100 (6/6)
**FI-EV71**	5 x 10^5^	0 (0/5)

hSCARB2-Tg mice were pre-immunized twice s.c. with PBS, 3 × 10^7^ pfu Ad-LacZ, 3 × 10^7^ pfu Ad-EVVLP, or 1 μg FI-EV71 vaccine on Days 1 and 7 after birth prior to being challenged s.c. with 5 × 10^5^ or 3 × 10^6^ pfu CVA16.

*Number of surviving mice per total number of tested mice was shown, and the survival rate was calculated.

## Discussion

In previous studies, EV71 subunit vaccines including DNA vaccine and recombinant VP1 protein induced an incomplete immune response and showed lower efficacy [27,41]. Oral vaccines, such as those against attenuated *Salmonella enterica* expressing EV71 VP1, have demonstrated limited efficacy against EV71, elevating the survival rates to only 50% after viral challenge [42]. Transgenic tomatoes [43] and peptide vaccines [44] expressing VP1 have also been developed, but the vaccine efficacy has not been assessed *in vivo*. A denatured virus particle containing formalin as a vaccine (FI-EV71) was tested in a hSCARB2-Tg mice model [16] and in human clinical trials [38], in which its safety and protective efficacy was demonstrated. A previous study on the development of influenza VLP as a vaccine showed that disrupting the influenza VLP structure abolished humoral immune responses and protective immunity [45]. In addition, the denatured EV71 particle possesses linear epitopes to elicit anti-EV71 antibodies; however, most of them are likely to be nonneutralizing, similar to the case of poliovirus [46]. Loss of the induction of effective neutralizing antibodies may be associated with the loss of antigenic determinants during inactivation, such as denatured EV71 particles by formalin. VLPs expressed in insect cells elicited even lower levels of neutralizing antibody titer, proliferation, and cytokine production in monkeys [47]. This may be due to differential post-translational modification of VLP proteins in nonhuman cells to induce differential immune responses. In contrast, intact VLPs produced from host cells preserve conformation-dependent epitopes, which might enable direct interaction of VLPs with B-cell receptors, activating B cells and antigen internalization through antigen-presenting cells [48]. This triggers potent antibody responses [49] and cross switching through cooperation with stimulated CD4^+^ T cells [50]. Furthermore, recent studies have shown that neutralizing antibodies, specifically those against the EV71 capsid proteins, cannot cross-protect against CV infection [19,21], indicating that the vaccines currently being developed protect against only EV71-induced HFMD.

In this study, we evaluated the potential of adenovirus-expressing EV71 VLP as a vaccine candidate against EV71 and CVA16 infections through comparison with the efficacies and immune responses elicited by Ad-EVVLP and the classical preparation of formalin-inactivated EV71 vaccine. Immunization with Ad-LacZ elicited no EV71-specific antibody titers and low levels of T cell responses, compared to Ad-EVVLP and FI-EV71 vaccines, which strongly induced the anti-EV71 antibody titer (Fig 3, Table 1). Antibodies induced by Ad-EVVLP exhibited cross reactivity against the clinically isolated EV71 C2 and B4 genotypes (Fig 3). In addition to anti-VLP antibody, the Ad-EVVLP vaccination induced anti-3C antibody (Fig 7). However, we did not observe the neutralizing activity against CVA16 in the serum of Ad-EVVLP- and FI-EV71-immunized mice (Table 1). This may explain why the anti-3C antibody could not bind to the 3C protein, which was either not expressed or was in the EV71 or CV inner capsid.

Previous studies have shown that preexisting anti-adenovirus antibodies do not affect subsequent generations of humoral responses to an antigen expressed through a mucosally administered recombinant adenovirus vector [24,25,51]. However, Ad-EVVLP oral immunization induced a decreased immune response compared to the mice receiving systemic Ad-EVVLP immunization (s.c. or i.p.; Fig 3 and Table 1). Our results showed that the existence of low anti-Ad antibody in sera of vaccine-primed animals (Fig D in S1 Text) did not influence the secondary VLP-specific antibody in the sera of mice administered a second dose of Ad-EVVLP orally, s.c., or i.p. The actual immuno-efficacy of Ad-EVVLP still needs to be assessed in clinical trials.

Ad is a strong DC activator, which enzymatically processes and presents antigenic peptides associated with MHC class I and II molecules on the surface, and subsequently coordinates and stimulates T helper and cytotoxic T-cell responses [52]. Ad-EVVLP immunization induced capsid protein-specific cellular immune responses, which was confirmed by the EV71 VLP induction of CD4^+^ and CD8^+^ T cell activation (Fig 5) and cytokine production (Fig 4). Compared to FI-EV71 vaccine immunization that activated Th2-mediated responses [16] associated with IL-4 and IL-13 secretion (Fig 4), the high IFN-ɣ, IL-4 and IL-13 levels produced by Ad-EVVLP-immunized splenocytes (Fig 4) suggested a mixed Th1/Th2 immune response, which potentiates both the activation of effector cellular responses and antibody production. These results are consistent with the induction of Th1/Th2 immune responses from the VLP of the influenza virus [45] and human papillomavirus [53]. Conversely, the CD4^+^ and CD8+ T cell activation corresponding to VLP was not observed in the FI-EV71-immunized mice (Fig 5), indicating that the epitopic antigenicity of VLP in the FI-EV71 vaccine after formalin inactivation was changed from its native form of EV71 VLP. However, structural analysis has shown that FI-EV71 is not different from infectious EV71 virions [13], and immunogenicity studies have revealed that the formalin-inactivated F- and E-particles of EV71 can induce the neutralizing antibody, even though the F-particle was more potent than E-particles in mice [37]. Thus, the antigenicity of the Ad-EVVLP-expressed VLP compared to the FI-EV71 vaccine VLP in the activation of cellular responses will be investigated in the future.

CD4^+^ and CD8^+^ T cell-mediated cellular responses corresponding to the recombinant 3C protein in Ad-EVVLP- but not FI-EV71 vaccine-immunized mice was also observed (Fig 7). We demonstrated that Ad-EVVLP immunization fully protected hSCARB2-Tg mice against EV71 (Fig 6) and CVA16 challenge (Fig 8 and Table 2). These results suggest that protection against EV71 infection through Ad-EVVLP is mediated by the induction of EV71-VLP-specific neutralizing antibodies, as well as VLP- and 3C-specific cellular immunities. The lower titer of neutralizing antibodies accompanied by higher transmission rates in children and infants indicates that neutralizing antibodies are crucial for the prevention of EV71 infection [54,55]. Our study also demonstrated that challenge of hSCARB2-Tg mice with EV71 followed by VP1 specific monoclonal antibody treatment might prevent EV71-induced pathology [19]. However, serum in 80% of EV71-infected patients contain neutralizing antibodies 1 day after illness onset; the level of antibody titer does not correlate with disease severity [56]. In contrast, cellular immune responses correlate with disease progression and clinical outcome [39,57]. Decreased cellular immunity is associated with increased disease severity in EV71 patients, whereas neutralizing antibodies display no difference between mild and severe cases [40]. These studies suggest that cellular immunity might be crucial in the protection against enterovirus infection. Our results showed that the 3C-specific cellular immunity induced by Ad-EVVLP might be sufficient to protect against CVA16 infection (Fig 8 and Table 2) even though Ad-EVVLP did not induce a CVA16-VLP-specific neutralizing antibody (Table 1). Therefore, we constructed Ad-3CD only expressing the 3CD gene and immunized hSCARB2-Tg mice followed by EV71 or CVA16 challenge. The results showed that Ad-3CD fully protected animals from EV71 and CVA16 challenges (Fig E in S1 Text). They indicate that 3CD-specific cellular immunities are sufficient to provide protection against EV71 and CVA16 infections. Therefore, we will further examine 3C and/or 3D-specific immune responses induced by the Ad-3CD vaccine and the identification of CD4 and CD8 epitopes in 3C and 3D proteins.

In conclusion, VLP expression in host cells through the replication of defective adenovirus mimicking the natural structure of EV71 particles induced antibodies against VLP and 3C proteins and cellular immunities specific to VLP and 3C proteins. Because the 3C protein is highly conserved between EV71 and CVA (Table A in S1 Text), we demonstrated that Ad-EVVLP acts as a multivalent vaccine to suppress EV71 and CVA16-induced disease. We achieved several breakthroughs in the development of a medically necessary enterovirus vaccine. First, instead of the subunit EV71 vaccine, inactivated EV71 vaccine, or protein-typed VLPs that protect against only EV71-induced HFMD, Ad-EVVLP prevents EV71- and CVA-induced HFMD. Second, induction of 3C-specific cellular immunity might sufficiently protect against CVA infection.

## Supporting Information

S1 Text The supporting information including Table A and Figs A to E were shown. (DOCX)
