# Supplementary material for: Recombinant Adeno-Vaccine Expressing Enterovirus 71-Like Particles against Hand, Foot, and Mouth Disease
Source: PLoS Negl Trop Dis. 2015 Apr 9;9(4):e0003692. doi: 10.1371/journal.pntd.0003692 (PMC4391779; doi:10.1371/journal.pntd.0003692)
Supplement: S1 Text — (DOCX) [file pntd.0003692.s001.docx]

**Supporting Information:**

**Table A.** Alignment of amino acid sequences within the EV71 3C and 3D regions of different CV strains.

**
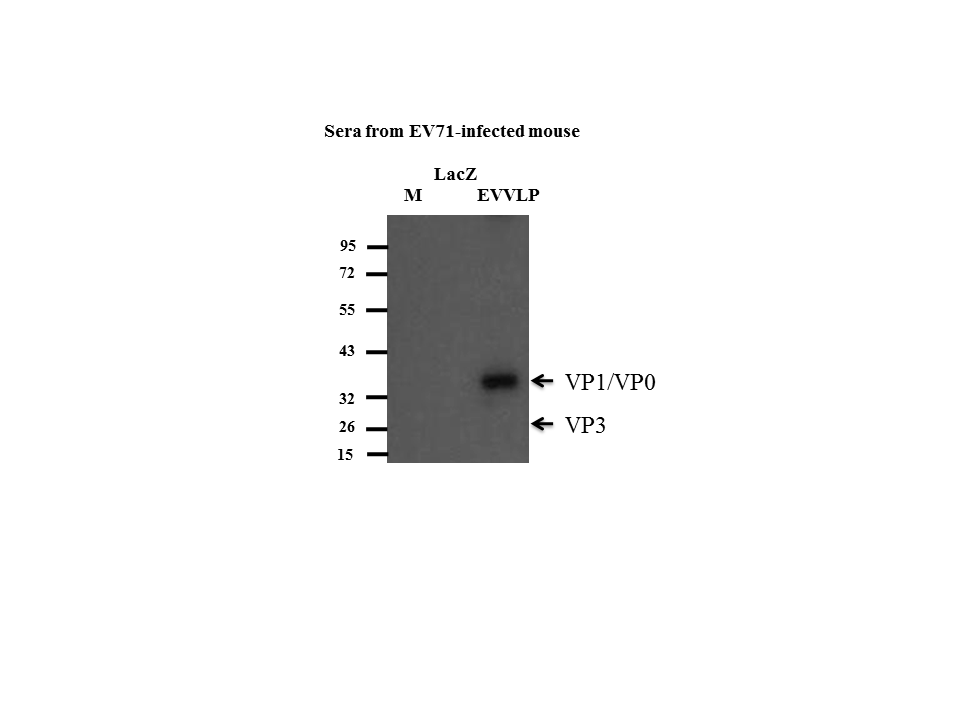
**

**Figure A.** Immunoblotting of VLP expression in Ad-EVVLP-infected cells with serum from EV71-infected mice.

The lysates of Ad-LacZ- and Ad-EVVLP-infected 293A cells were analyzed by immunoblotting with polyclonal serum from BALB/c mice i.p. injected with 10^6^ pfu EV71 5746. The protein marker (M) is marked.

**Figure B.** Enumeration of IFN-γ and IL-4-secreting cells in spleens of Ad-EVVLP-immunized mice.

Splenocytes prepared from mice immunized twice with Ad-LacZ or Ad-EVVLP i.p. or s.c. were cultured and supplemented with murine IL-2 in the presence of UV-EV71 E59 in anti-IFN-γ (left panel) or anti-IL-4 (right panel) capture antibody-coated wells of an ELISPOT plate for 2 days. Cytokine-positive immunospots were developed using the reagents and protocol provided in the assay kit. The results are expressed as the number of specific cytokine immunospots ± 2 standard deviations for each group.

**
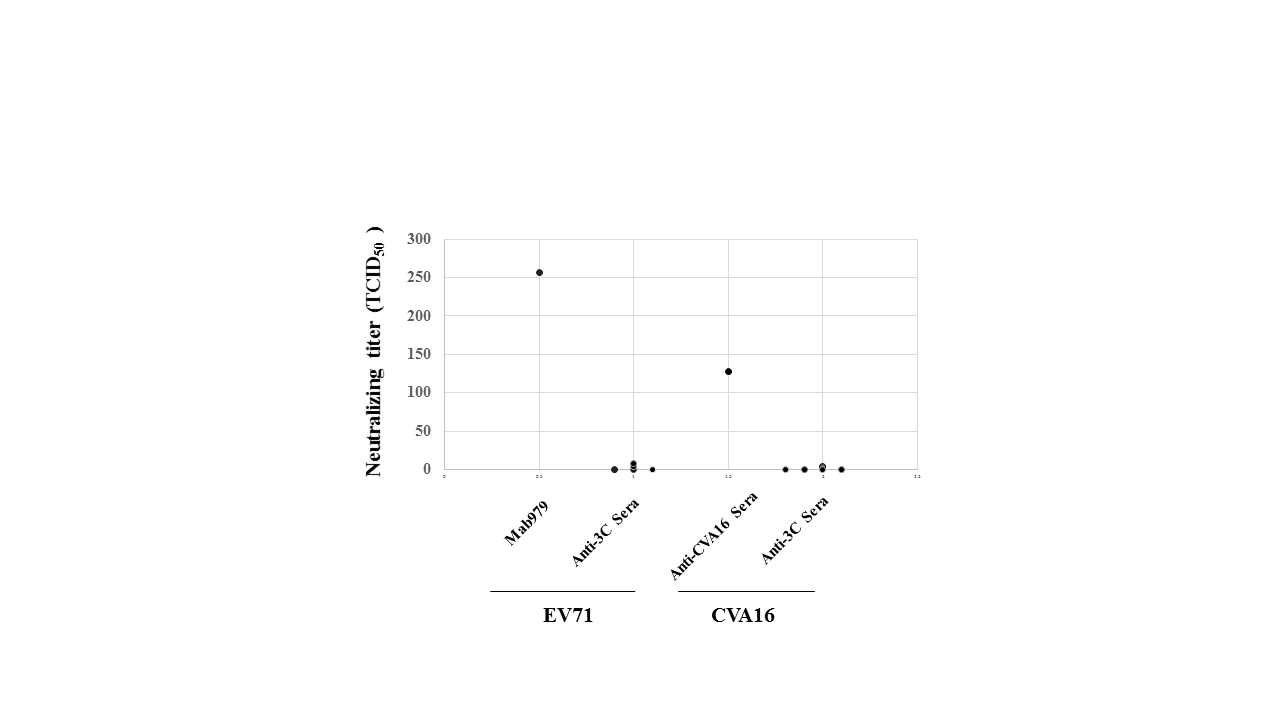
**

**Figure C.** Mouse sera from recombinant 3C adjuvanted with CFA/IFA do not elicit neutralizing activity against EV71 and CVA16.

Seven-week-old BALB/c mice were individually primed s.c. with 10 µg recombinant 3C adjuvanted with CFA (3C-CFA) and then s.c. boosted with the same dose of 3C-IFA at 14-day intervals. Sera collected on Day 21 were assayed for neutralizing activity by incubating 10^2^ pfu EV71 or CVA16 with varying dilutions of individual immune sera before being added to RD cells. CPE were observed after 5 days of culture. The results are expressed as neutralizing titers that correspond to the dilution of immune sera, giving TCID_50_ value of 50% reduction of cytopathic effect. Five mice per group were assayed. Varying dilutions of Mab979 antibody and serum collected from BALB/c mice infected with 10^6^ pfu CVA16 at 14 days post-infection for neutralizing EV71 and CVA16, respectively, were included as positive controls.

**
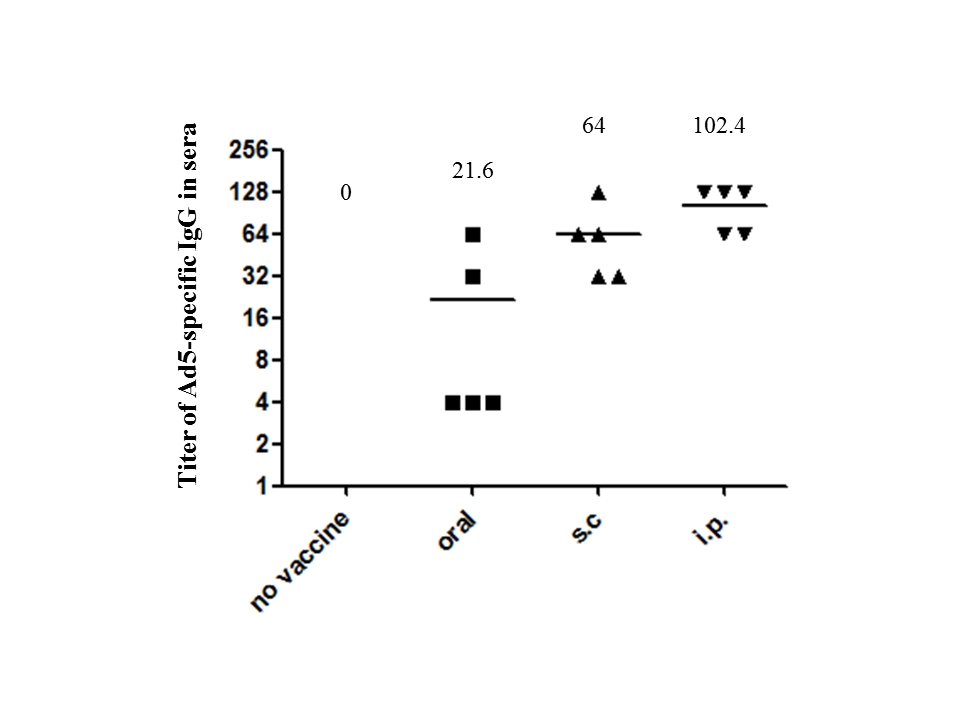
**

**Figure D.** Induction of Ad5-specific IgG in the serum of Ad-vaccinated mice.

Seven-week-old BALB/c mice were individually primed and boosted at 14-day intervals though oral, s.c., or i.p. routes with or without 10^8^ pfu Ad-EVVLP. Serum samples collected on Day 21 were assayed for IgG against heat-inactivated Ad5-immobilized ELISA. The results are expressed as titers for each test sample. Bars correspond to mean titers for each experimental group of 5 mice.


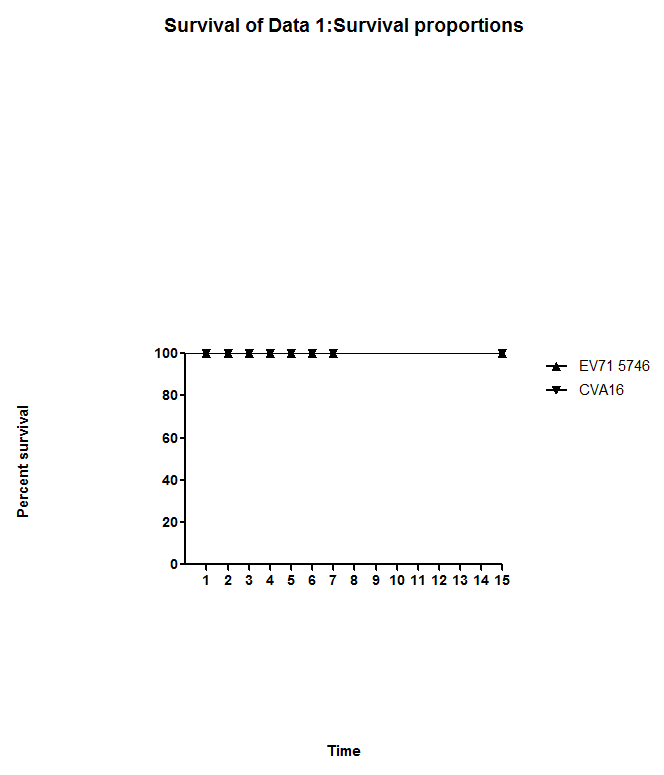


**EV71 (3X10^6^)**

**CVA16 (3X10^6^)**

**Percent survival (%)**

**Days post infection**

**N=**

**8**

**7**

**Figure E.** Ad-3CD protects hSCARB2-Tg mice from EV71 and CVA16 challenges.

One-day-old hSCARB2-Tg mice were pre-immunized twice s.c. with 3 × 10^7^ pfu Ad-3CD on Days 1 and 7 after birth prior to being challenged s.c. with 3 × 10^6^ pfu (▲) EV71 or (▼) CVA16. Control group immunized with 3 × 10^7^ pfu Ad-LacZ was performed and shown in Table 2. The survival of mice was monitored on a daily basis for 15 days. The number (N) of transgenic mice is shown.
